# HMGB1 induces radioresistance through PI3K/AKT/ATM pathway in esophageal squamous cell carcinoma

**DOI:** 10.1007/s11033-022-07989-8

**Published:** 2022-10-19

**Authors:** Xueyuan Zhang, Naiyi Zou, Wenzhao Deng, Chunyang Song, Ke Yan, Wenbin Shen, Shuchai Zhu

**Affiliations:** Department of Radiation Oncology, The Fourth Hospital of Hebei Medical University, 12 Jiankang Road, Shijiazhuang, Hebei 050011 People’s Republic of China

**Keywords:** ESCC, HMGB1, Radioresistance, Apoptosis, Cell cycle

## Abstract

**Background:**

To explore the effect of HMGB1 on the radio-sensitivity of esophageal cancer cells through regulating the PI3K/Akt/ATM pathway.

**Methods and results:**

We observed the expression of HMGB1 and p-ATM in biopsies of esophageal cancer patients with immunohistochemical staining. Western blot and RT-qPCR were applied to detect the protein and RNA related to PI3K/Akt/ATM pathway, respectively. In addition, we inhibited the PI3K/Akt pathway with ly294002 and activated it with IGF1, then we explored the invasion, proliferation ability, and apoptosis of esophageal cancer cells in vitro by transwell, CCK8 assay, and flow cytometry respectively. In vivo, xenograft tumor model was established in nude mice to study the effect of HMGB1 on radioresistance via PI3K/AKT/ATM Signaling Pathway. The survival rate in patients with single positive/double negative expression of HMGB1 and p-ATM was significantly higher than in those with both positive expression of HMGB1 and p-ATM, the depletion of HMGB1 combined with ly294002 significantly inhibited cell proliferation and invasion ability, meanwhile, the addition of IGF1 reversed it. Meanwhile, depletion of HMGB1 and ly294002 promoted apoptosis and arrested the cancer cells in G0/G1 cell cycle with the decreased expression of Cyclin D1 and CDK4 and improved P16. We further validated these results in vivo, the application of HMGB1 silencing promoted apoptosis of xenograft tumors after radiation, especially combined with pathway inhibitor ly294002.

**Conclusions:**

Esophageal cancer patients with high expression of HMGB1 and p-ATM have a poor prognosis after chemo-radiotherapy. Down-regulation of HMGB1 may promote the radio-sensitivity of esophageal cancer cells through regulating PI3K/Akt/ATM pathway.

## Introduction

According to cancer statistics in 2020, esophageal cancer ranks 7th and 6th in the incidence and mortality of malignant tumors in the world respectively [1]. Due to the differences in environmental factors, lifestyle, and gene types, esophageal cancer has obvious regional characteristics with different pathological types. Squamous cell carcinoma is the main pathological type of esophageal carcinoma in China, Japan, Korea, Kazakhstan, Turkey, and northeastern Iran [2, 3]. Early stage esophageal cancers are unnoticeable leading to most patients missing the operation for a progressive stage, radiotherapy has become the main treatment for patients with advanced esophageal cancer [4–6]. In clinical practice patients with the same lesion site and same stage of esophageal cancer often receive the same radiotherapy regimen, some patients’ pathological responses can achieve CR (complete response), while other patients only have SD (stable disease) or PD (progressive disease) [7]. This may be accounted for the difference in radio-sensitivity due to the heterogeneity of tumor tissues among different patients. Therefore, the exploration of molecular markers related to the radio-sensitivity of esophageal squamous cell carcinoma cells and the further clarification of its internal regulation mechanism of radio-sensitivity can provide a new idea and direction for the treatment of esophageal squamous cell carcinoma.

Ionizing radiation kills tumor cells mainly by causing DNA damage. After exposure to radiation, tumor cells will activate a series of stress factors to produce a DNA repair response to maintain cell proliferation and inhibit apoptosis, resulting in radio-resistance [8]. Therefore, DNA damage repair ability is the key reason for radiation resistance. HMGB1 is a non-histone chromosome binding protein in mammalian cells in large quantities [9]. Through acetylation, HMGB1 binds to the corresponding DNA polymerase—to form a specific complex, thus activating its biological functions which include regulating DNA replication, repair and transcription [10]. Therefore, HMGB1 may be involved in the generation of tumor radio-resistance. And HMGB1 plays an important role in effecting the biological behavior of breast cancer cells by regulating the PI3K/Akt pathway [11]. Previous studies indicates that the P13K/Akt pathway is involved in the malingnant behavior of cancerous cells [12]. And the application of PI3K pathway inhibitors can improve the sensitivity of tumor cells to different therapeutic treatment modalities, such as chemotherapy, radiotherapy [12] and targeted therapies [13]. In this study, we aim to detect the internal mechanism of HMGB1 affecting the radio-sensitivity of esophageal cancer cells with PI3K/Akt pathway inhibitor and promoter.

## Method

### Immunohistochemical (IHC) staining

Immunohistochemistry was performed as previously described [14]. From January 2008 to December 2013, a total of sixty-one endoscopic biopsy specimens of esophageal squamous carcinoma patients who received chemo-radiotherapy (CRT) were obtained in the fourth Hospital of Hebei Medical University. We acquired the approval from the Ethics Committee of the fourth Hospital of HeBei medical university and informed consent was obtained from all subjects and/or their legal guardians. According to the scoring standard of previous studies [15], two blinded, independent pathologists simultaneously examined HMGB1 and p-ATM staining, and each core reached a consensus score.

### Cell line culture

KYSE30 and ECA109 human esophageal cancer cell lines were maintained in RPMI-1640 (Gibco, USA) supplemented with 10% FBS (FBS, Invitrogen, USA) and antibiotics (100 units/ml penicillin and 0.1 mg/ml streptomycin, Gibco, USA) in Thermo Scientific CO_2_ incubators with the temperature set at 37 °C and the concentration of carbon dioxide set to 5%. All cells were proved from the Scientific Research Center of the fourth Hospital of Hebei Medical University.

Ly294002, PI3-K inhibitor, was obtained from MCE, MedChemExpress, USA. It is dissolved in DMSO at a stock concentration of 10 mM. In the cell experiment, the working concentration of ly294002 was 10 μM. The mice were treated with 50 mg/kg of ly294002 diluted with PBS, via an i.p. injection, twice weekly, for 3 weeks. The rest of the mice received the same amount of vehicles only.

IGF-1, PI3-K promoter, was obtained from Sino Biological (Sino Biological, Inc., Beijing) and stored at −20 °C. After reconstitution with ddH_2_O, the working solution of IGF-1 was 10 ng/ml in the cell experiment.

### Cell transfection

Cell transfection was performed as previously described [14] with human HMGB1 shRNA and negative control shRNA (Public Protein/Plasmid Library, China) using Lipofectamine 2000 (Invitrogen, USA) under the guidance of the manufacturer's instructions, and recorded as HMGB1-shRNA group and NC group respectively. The concentrated virus solution and esophageal cancer cells were cocultured, and stable cell lines with the fluorescence were observed by optical microscopy and identified by Western blotting and RT-qPCR. Untreated group of cancer cells were considered as control group.

### Quantitative real-time reverse transcription polymerase chain reaction

RNA was extracted from esophageal tumor cells with TRIzol reagent (TaKaRa, Japan) and reversed transcribed to cDNA with a RevertAid First Strand cDNA Synthesis Kit (Thermo, USA). Then, RT-PCR was applied to analyze the expression of the HMGB1 gene using Platinum SYBR-Green qPCR SuperMix-UDG (Invitrogen, USA) under the guidance of the reagent’s instructions. The GAPDH gene was used for the normalization of RT-qPCR data. The 2^−ΔΔCt^ method was applied to analyze HMGB1 gene expression.

### Western blot analysis

We conducted Western blot analysis following the method described previously [16]. The relevant antibodies were anti-HMGB1 (1:10,000; Abcam, USA), anti-Akt(AKT3 + AKT2 + AKT1,1:5000; Abcam) anti-Akt (phosphor S473, 1:5,000; Abcam), anti-PI3K p110(1:1000; Abcam), anti-Ki67 (1:1,000; Abcam), anti-ATM (1:2,000; Abcam), anti-ATM (phosphor Ser1981, 1:1,000; Novus), anti-P16(1:1,000; Proteintech, Chicago, USA), anti-CDK4 (1:2,000; Proteintech), anti-cyclin D1(1:5000; Proteintech) and anti-β-actin (1:10,000; Bioworld Technology Inc. USA), phosphatase inhibitor cocktail III(1:100; MCE, MedChemExpress, USA). The blotted protein bands were revealed by an Odyssey system (LI-COR Biosciences, USA). Finally, we measured the intensity of protein bands with ImageJ and calculated the ratio of the protein to the corresponding β-actin for the purpose of reflecting the changes in expression levels.

### Cell proliferation assay

We use CCK-8 kit (MCE, MedChemExpress, USA) to detect the proliferation ability of tumor cells. Making cell suspensions up to 5 × 10^4^ cells/ml, take 100 μl into 96-well plates with five duplicates for each sample. At certain time points, incubating the cells with 10 μl CCK-8 for 2 h. Then, detecting the absorbance of each well at 450 nm via a Multiskan. The experiment was repeated at least three times.

### Transwell assay

The invasion ability of tumor cells was detected using transwell chambers (Corning Costar, USA) coated with matrigel (Corning, USA) before use. After washing with PBS, cells were diluted to 5 × 10^5^/ml with serum-free medium and transferred 200 μl onto the top of the chambers. Medium containing 10% fetal bovine serum was put into the lower chamber. After penetrating for 24 h, we swept cells on the upper side of the chamber slightly. Meanwhile, the cells passing through the filter were fixed with formaldehyde and stained with crystal violet for 10 min. Then we photographed and counted the number of stained cells with a microscope from 5 randomly selected fields (magnification, × 200).

### Cell cycle and apoptosis

We detected cell apoptosis with an Annexin V-FITC apoptosis detection kit (BD Biosciences). In accordance with the kit’s instructions, cells were collected and stained with propidium iodide (PI) and Annexin V-FITC. Flow cytometry was applied to detect the cell cycle distribution and apoptosis rates, encompassing both the early apoptosis (annexin V + /PI−) and late apoptosis/necrosis (annexin V + /PI +) phases.

### Xenograft tumor models

Twenty-four male BALB/c nude mice were obtained from Beijing Vital River Laboratory Animal Technology Co., Ltd. (Beijing, China). All experiments with animals were carried out with the approval of the Animal Care and Use Committee of the fourth Hospital of Hebei Medical University, and the use of animals in experiments was observed by the Interdisciplinary Principles and Guidelines for the Use of Animals in Research, Testing, and Education by the New York Academy of Sciences, Ad Hoc Animal Research Committee. A total of 4 × 10^6^ HMGB1-shRNA or NC tumor cells were injected into the left hind paw of the mice. Two weeks after injection, irradiation (5 Gy) was performed each day for a total of 3 days with a collimator container to protect the normal tissue. Calipers was used to measure the tumor diameter (mm) twice a week, and the tumor volume (TV) was calculated by the following formula: TV = AB^2^/2, where A represents the tumor’s long diameter and B represents the short diameter.

### Statistical analysis

The data collected were analyzed using the SPSS software package version 21 (SPSS, Inc., Chicago, IL, USA) and recorded as the mean ± standard deviation (SD). The endpoint of progression-free survival (PFS) was measured from the beginning of CRT to progression, death or the date of the last follow-up. The survival analysis was carried out by Kaplan–Meier method with log-rank test and the χ2 test or Fisher’s exact test was used to analyze the association between clinical parameters and HMGB1 or p-ATM expression. The comparisons of data between different groups were made by ANOVA least significant difference test. If the p value of a two-sided statistic test was lower than 0.05 or 0.01, we considered the result to be statistically significant.

## Results

### Association between the clinical characteristics and expression level of HMGB1 and p-ATM protein

**Fig. 1:**
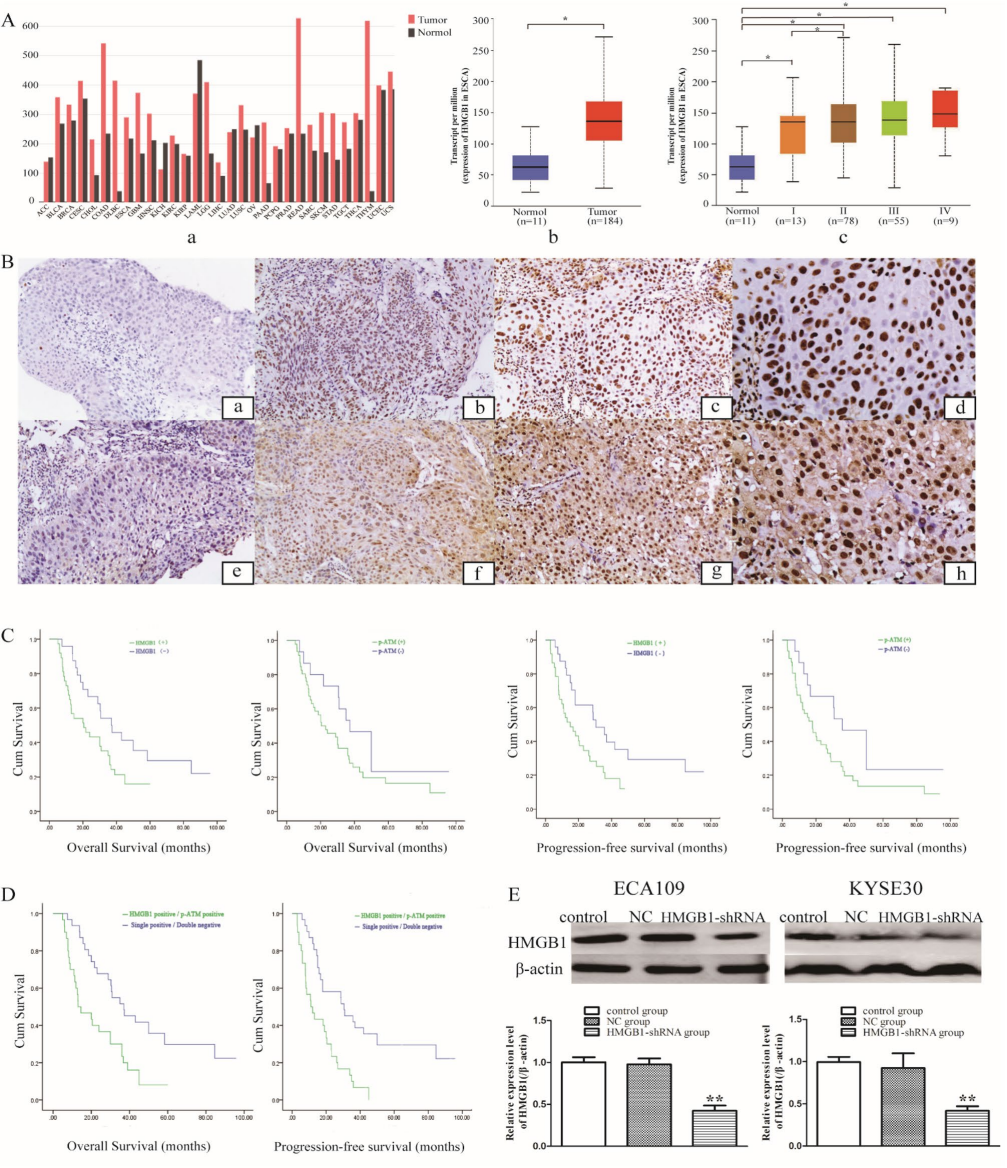
The relationship between the survival and the expression of HMGB1 and p-ATM of ESCC patients **A**. Data were obtained from TCGA database. The expression of HMGB1 in a variety of tumors (*a*). The expression of HMGB1 was high in ESCA compared with normal tissues (*b*). The expression of HMGB1 in different stage of ESCA. Immunohistochemical staining **B** showed the expression of HMGB1 and p-ATM in ESCC samples was higher (*a* and *e*, adjacent tissues, magnification × 100; *b* and *f*, weakly positive, magnification × 100; *c* and *g*, positive, magnification × 200; d and h, strongly positive, magnification × 400). **C**. Overall survival and progression-free survival among negative and positive expression of HMGB1 and p-ATM, respectively. **D**. The overall survival and progression-free survival in patients with single positive/double negative expression of HMGB1 and p-ATM compared with both positive expression of HMGB1 and p-ATM. E. the suppression of HMGB1 protein and mRNA expression was observed in the HMGB1-shRNA group by Western blot analysis and RT-qPCR analysis. Cells were transfected with specific human HMGB1 shRNA (HMGB1-shRNA group) or the negative control shRNA (NC group) or without any transfection (control group). *p < 0.01 **p < 0.01.

Association between the clinical characteristics and expression level of HMGB1 and p-ATM protein was analyzed. Table 1 revealed that the expression of HMGB1 and p-ATM have no association with sex, age, lesion location, or N classification (P > 0.05). Nonetheless, the expression of HMGB1 in esophageal cancer was significantly associated with gross tumor volume (GTV) (P = 0.014) and TNM stage (7th AJCC) (P = 0.028). In addition, the expression of p-ATM in esophageal cancer was significantly associated with TNM stage (P < 0.001), T classification (P = 0.008) and relapse (P = 0.045). Combined HMGB1 and p-ATM, the results showed patients with both positive expression of HMGB1 and p-ATM have larger tumor and more distant metastasis than those with single positive/double negative expression of HMGB1 and p-ATM (Table 2). Through online analysis of UALCAN database, we obtained the expression of HMGB1 in a variety of tumors (Fig. 1Aa). The expression of HMGB1 was high in ESCA compared with normal tissues (Fig. 1Ab) and tumors with earlier stage tended to have lower gene expression of HMGB1 (Fig. 1Ac), data were obtained from TCGA database. According to the results of immunohistochemical staining, the expression of HMGB1 (Fig. 1B(b–d)) and p-ATM (Fig. 1B(f–h)) in ESCC samples was higher in comparison with adjacent tissues with no tumor complication (Fig. 1B(a–e)). Survival analysis indicated that the HMGB1 expression level in ESCC were significantly associated with overall survival (χ^2^ = 4.770, P = 0.029; Fig. 1C), but no association was found between p-ATM and OS (χ^2^ = 2.874, P = 0.090; Fig. 1C). Meanwhile, both HMGB1 and p-ATM expression levels were significantly associated with progression-free survival (P = 0.023, Fig. 1C; P = 0.033, Fig. 1C). The survival of patients with positive expression of HMGB1 or p-ATM were significantly shorter compared with patients with negative expression of HMGB1 or p-ATM according to the results of the log-rank test. In addition, the overall survival and progression-free survival in patients with single positive/double negative expression of HMGB1 and p-ATM was significantly better than those with both positive expression of HMGB1 and p-ATM (P < 0.001, Fig. 1D). In summary, statistical analysis revealed that HMGB1 and p-ATM overexpression is associated with more aggressive tumors, resistance to ionizing radiation and poor prognosis. The combination of HMGB1 and p-ATM expression can better predict the prognosis of patients with esophageal cancer after chemo-radiotherapy.

**Table 1:** Association between the clinical characteristics and expression of HMGB1 and p-ATM protein

Characteristics	n	HMGB1		P-value	p-ATM		P-value
		Negative	Positive		Negative	Positive	
Age (years)							
≤ 67	30	10	20	0.344	7	23	0.823
> 67	31	14	17		8	23	
Sex							
Male	39	13	26	0.739	8	31	0.325
Female	22	11	11		7	15	
Gross tumor volume (GTV)							
≤ 30	24	14	10	0.014	9	15	0.059
> 30	37	10	27		6	31	
Lesion location							
Neck/upper	23	10	13	0.607	5	18	0.687
Middle/ lower	38	14	24		10	28	
TNM stage^a^							
I-II	30	16	14	0.028	13	17	P < 0.001
III-IV	31	8	23		2	29	
T classification							
T1-T2	23	11	12	0.291	10	13	0.008
T3-T4	38	13	25		5	33	
N classification							
N0	25	11	14	0.535	7	18	0.606
N1-N3	36	13	23		8	28	
Distant metastasis							
M0	12	7	5	0.241	6	6	0.057
M1	49	17	32		9	40	
Relapse							
Negative	31	13	18	0.674	11	20	0.045
Positive	30	11	19		4	26	

^a^All cases were classified into clinical TNM stage according to the criteria of the UICC and the AJCC in 2009. *UICC* international union against cancer; *AJCC* american joint committee on cancer; *HMGB1* high mobility group box 1; *TNM* tumor-node-metastasis

**Table 2:** Association between the clinical characteristics and combined expression of HMGB1 and p-ATM

Characteristics	n	HMGB1 and p-ATM		P-value
		Single positive/double Negative	HMGB1 Positive/p-ATM positive	
Age (years)				
≤ 67	30	14	16	0.612
> 67	31	17	14	
Sex				
Male	39	16	23	0.062
Female	22	15	7	
Gross tumor volume (GTV)				
≤ 30	24	17	7	0.018
> 30	37	14	23	
Lesion location				
Neck/upper	23	12	11	0.869
Middle/ lower	38	19	19	
TNM stage				
I-II	30	21	9	0.005
III-IV	31	10	21	
T classification				
T1-T2	23	16	7	0.034
T3-T4	38	15	23	
N classification				
N0	25	`15	10	0.766
N1-N3	36	16	20	
Distant metastasis				
Negative	12	9	2	0.022
Positive	49	22	28	
Relapse				
Negative	31	18	13	0.310
Positive	30	13	17

### Effects of HMGB1 on proliferation and invasion ability in esophageal cancer cells by targeting PI3K/Akt/ATM pathway

**Fig. 2:**
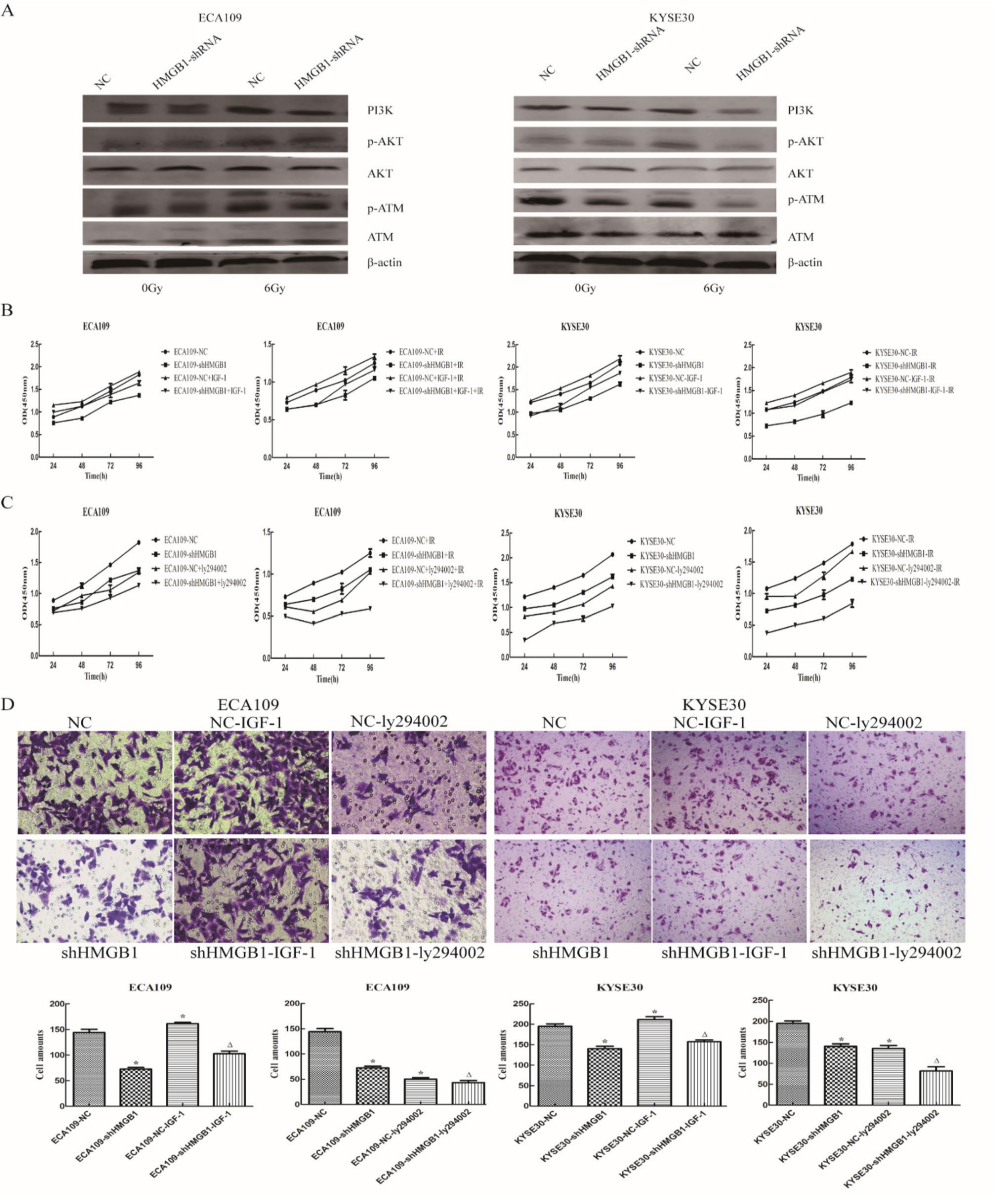
Downregulation of HMGB1 inhibited proliferation and invasion ability of ESCC by targeting PI3K/Akt/ATM pathway Proteins were extracted 1 h after irradiation. The results of western blot showed that downregulation of HMGB1 decreased the expression of PI3K, p-Akt and p-ATM compared with corresponding NC group after irradiation, no difference was found in Akt and ATM expressions in ECA109 and TE13 cell lines. The results of CCK8 assay showed that depletion of HMGB1, ly294002 and radiation inhibited the cell proliferation **C**, but the addition of IGF1 enhanced the proliferation ability and suppress the weaker effect induced by HMGB1 depression in ECA109 and KYSE30(B). **D**. The results of Transwell assay showed that silencing HMGB1 and applying ly294002 could reduce the number of the cells passing through the filter, and IGF1 can increase the number of the cells and weaken the effect of reducing the invasion ability caused by silencing HMGB1. *p < 0.05; a comparison with the corresponding no pathway intervention group is symbolized by a triangle, ▲p < 0.05.

The effects of HMGB1 depression on the expression level of protein related to PI3K/Akt/ATM pathway was detected by western blot assay. And the results indicated downregulation of HMGB1 decreased the expression of PI3K, p-Akt and p-ATM compared with corresponding NC group after radiation, no difference was found in Akt and ATM expression between HMGB1-shRNA and NC group in ECA109 and TE13 cell lines (Fig. 2A). In addition, CCK8 assay was applied to detect the effect of the alteration of HMGB1 expression, inhibition and activation of PI3K/Akt pathway on the proliferation of ECA109 and TE13 cells. The cell growth curve showed that depletion of HMGB1, ly294002 and radiation inhibited the cell proliferation and the combined effect of three agents was more effective than single one (Fig. 2C). Meanwhile, the addition of PI3K/Akt pathway activator IGF1 enhanced the proliferation of esophageal cancer cells and suppress the weaker effect induced by HMGB1 depression (Fig. 2B). After irradiation, the proliferation activity significantly decreased compared with corresponding non-irradiated group. In terms of cell invasion ability, the results of Transwell assay suggesting that silencing HMGB1 and applying pathway inhibitor ly294002 could reduce the number of the cells passing through the filter, and the combined effect was more effective. However, the application of pathway activator IGF1 can increase the invasion ability and weaken the effect of reducing the invasion ability caused by silencing HMGB1 (Fig. 2D). These findings indicated that HMGB1 may regulate the proliferation and invasion ability of esophageal cancer cells through the PI3K/Akt/ATM signaling pathway.

### Effects of HMGB1 on apoptosis and the cell cycle in esophageal cancer cells via its targeting of PI3K/AKT/ATM signaling pathway

**Fig. 3:**
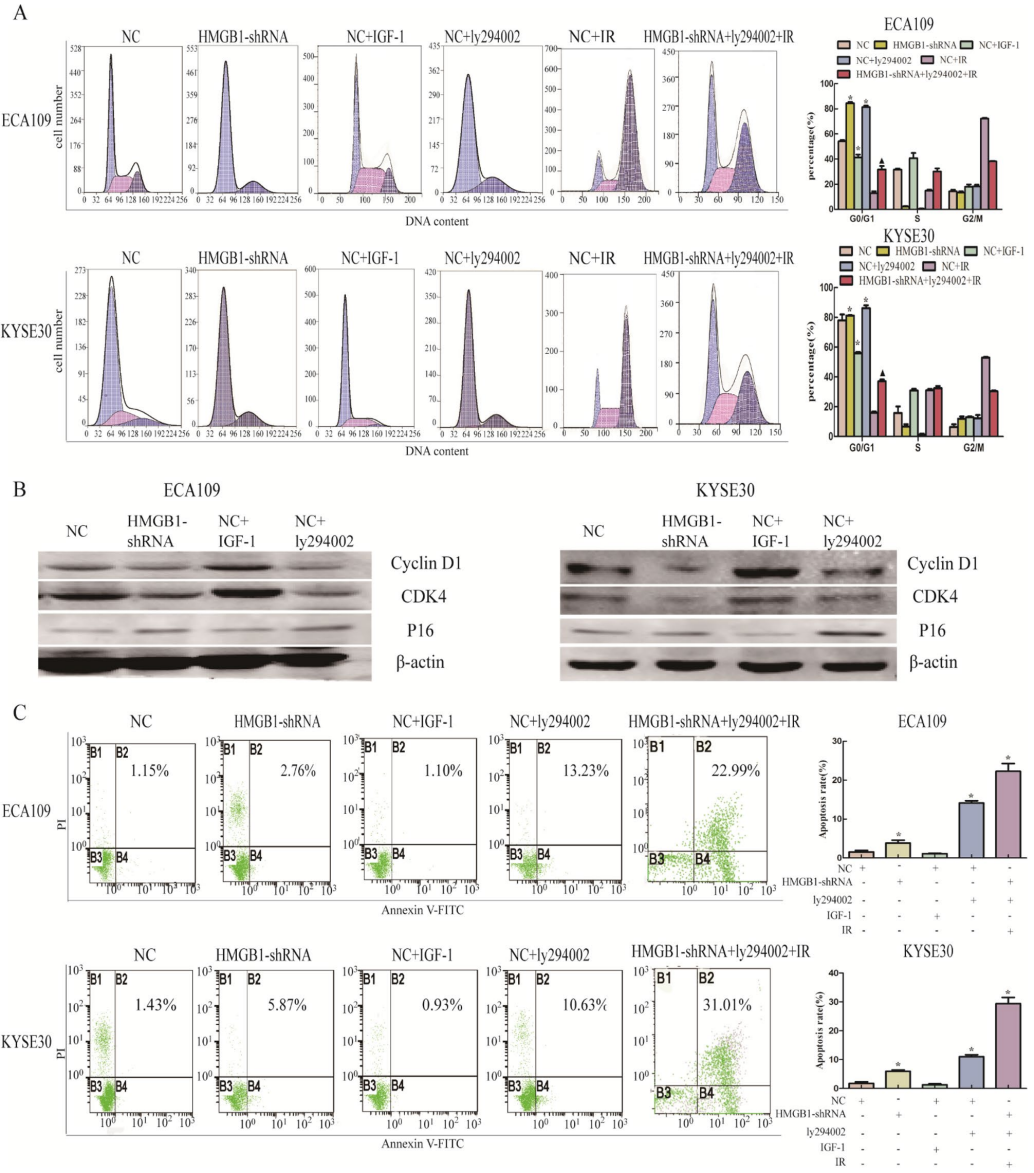
Downregulation of HMGB1 induced G0/G1 arrest and promoted apoptosis of ESCC through PI3K/AKT/ATM pathway The results of flow cytometry showed the percentages of G0/G1 phase cells in the HMGB1-shRNA group and NC + ly294002 group were significantly higher compared those of the NC group in ECA109 and KYSE30, and the percentages of G0/G1 phase cells were higher in HMGB1-shRNA + ly294002 + IR group compared with corresponding NC + IR group. In the HMGB1-shRNA group and NC + ly294002 group, the expression of cyclin D1 and CDK4 was attenuated, while the expression of P16 was increased **B**. We use B2 + B4 to calculate the apoptotic rate. As the results showed, compared with the NC group, silencing of HMGB1 and ly294002 sensitized ECA109 and KYSE30 cells to apoptosis both before and after irradiation **C**. *p < *0.05compared with NC group,* ▲p < *0.05 compared with NC* + *IR group.*

The results of flow cytometry showed that apoptosis levels in the ECA109-shRNA and KYSE30-shRNA groups were higher than those in the corresponding NC groups (P < 0.05), and the addition of IGF1 weak the effect of HMGB1 depression on apoptosis increasing of esophageal cancer cells (Fig. 3C), and the most significant apoptosis occurred in the depletion of HMGB1 combined with ly294002 and IR groups On the other hand, the depletion of HMGB1 arrested the cancer cells in G0/G1 cell cycle with the decrease expression of Cyclin D1 and CDK4 and improved P16, it has the same effect as the application of PI3K/Akt pathway inhibitors ly294002 (Fig. 3A–B). We next checked the effect of HMGB1 deficiency combined with ly294002 on cell cycle after treating the cells with 6 Gy irradiation, and the result demonstrated that the percentages of G0/G1 phase cells in the HMGB1-shRNA + ly294002 + IR groups were significantly higher compared those of the NC + IR group (Fig. 3A).

### Effects of HMGB1 on the growth of xenograft tumor

**Fig. 4:**
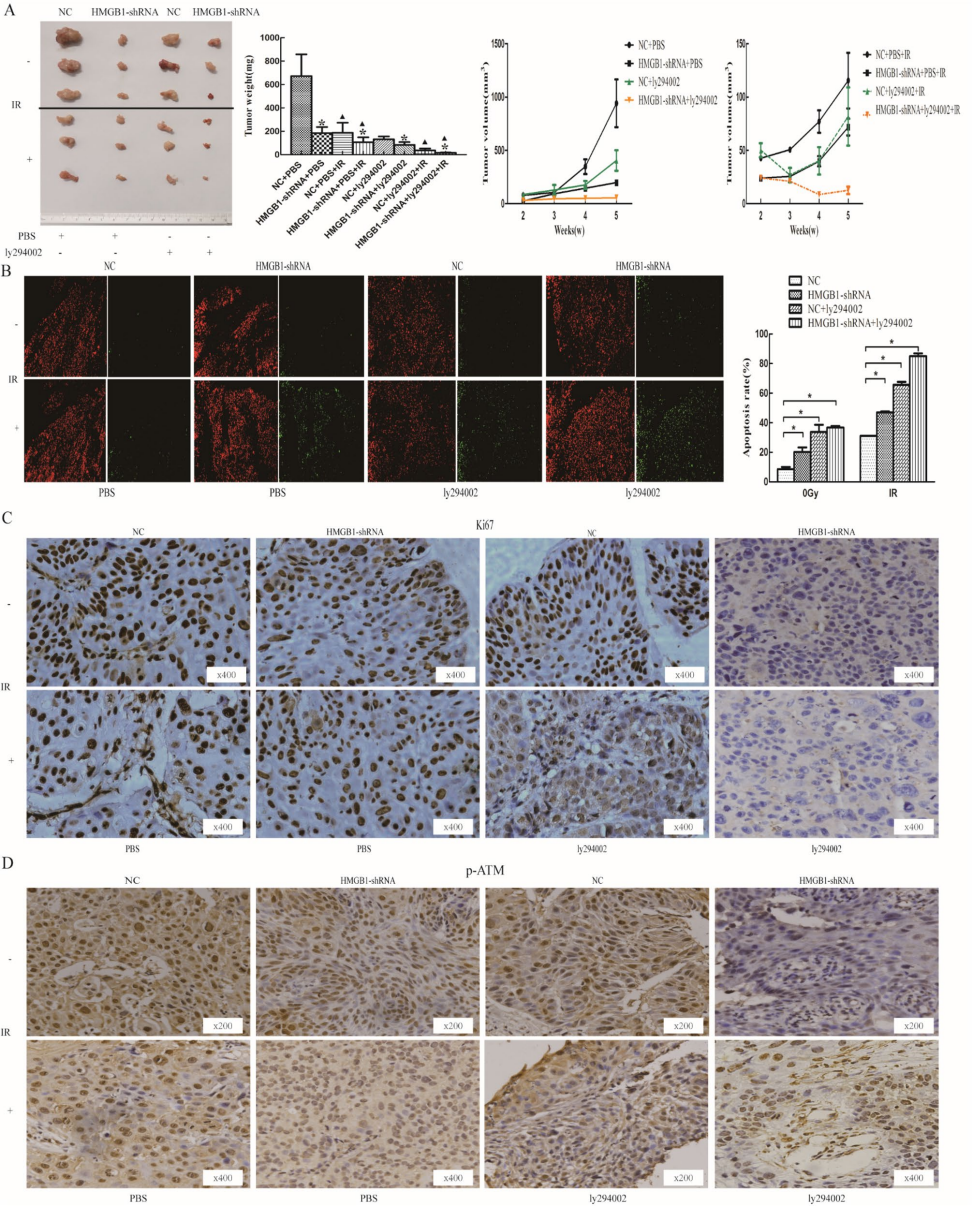
Downregulation of HMGB1 slowed down the growth and promoted apoptosis of xenografts The nude mice were sacrificed, the tumors were dissected, tumor weights and volume **A** were measured; the data are shown as the means ± SD. The growth rate of xenografts slowed down, the tumor volume and weight was reduced after the application of HMGB1 silencing, especially combined with pathway inhibitor ly294002, and the trend was more obvious after irradiation. TUNEL assay showed that downregulation of HMGB1 and ly294002 promoted the apoptosis of xenografts, especially combined with irradiation **B**. Using the immunohistochemistry method, the expression of Ki67 and p-ATM is higher in NC group compared with HMGB1-shRNA group, and the application of ly294002 further inhibited the expression of Ki67 **C** and p-ATM **D**. *p < 0.05; a comparison with the corresponding unirradiated group is symbolized by a triangle, ▲p < 0.05.

It was observed that the growth rate of xenografts in nude mice in the NC group treated with PBS was the fastest, and the growth rate of xenografts slowed down, the tumor volume and weight was reduced after the application of HMGB1 silencing, especially combined with pathway inhibitor ly294002, and the trend was more obvious after irradiation (Fig. 4A). The results of TUNEL assay showed that HMGB1 silencing promoted the apoptosis of xenografts after radiation (Fig. 4B). The expression of Ki67 and p-ATM is higher in NC group compared with HMGB1-shRNA group, and the application of ly294002 further inhibited the expression of Ki67 and p-ATM (Fig. 4C–D). It is shown that the silencing of HMGB1 can inhibit the growth of transplanted tumor, and it does play a radio-sensitization effect in vivo, and its mechanism is related to the PI3K/AKT/ATM signaling pathway.

## Discussion

Due to the differences in environmental factors, lifestyle, gene types and other aspects, the incidence of esophageal cancer has obvious regional characteristics and shows different pathological types [17]. The incidence of esophageal cancer in Henan, Hebei and Taihang mountains of China is about 100 times higher than the world average, and most of them are squamous cell carcinoma. Previous studies have shown that chemo-radiotherapy is the main treatment for advanced esophageal cancer patients who lose the chance of surgery, but the 5-year survival rate of radiotherapy alone is only about 20%, and local recurrence after radiotherapy is as high as 50% ~ 70% [18]. Increasing radio-sensitivity of tumor cells is an urgent problem to be solved for esophageal cancer patients.

Previous studies have shown that HMGB1 protein is involved in various tumor development processes, including proliferation [19], metastasis [11, 20], activation of multiple signaling pathways [21–23], DNA damage repair [24] and autophagy, et al. Simultaneously, evidence from other study suggests that HMGB1 knockdown increases the radio-sensitivity of esophageal squamous cell carcinoma by regulating the expression of nicotinamide adenine nucleotide phosphate oxidase-mediated ROS production and inducing DNA damage via the MAPK signaling pathway [25]. These results suggest that HMGB1 may be closely related to the radio-sensitivity of esophageal cancer. PI3K (phosphatidylinositol kinase) is a dimer composed of regulatory subunit P85 and catalytic subunit P110 [12]. When it binds to growth factor receptors, it changes the protein structure of Akt and activates it, and phosphorylates a series of downstream substrates, such as apoptosis-related proteins Bad and Caspase9, to regulate cell proliferation, differentiation, apoptosis and migration [26, 27]. We observed that downregulation of HMGB1 protein resulted in changes in p-AKT and p-ATM expression of esophageal cancer cells. So we speculate there are some relationship between HMGB1 and PI3K/Akt/ATM pathway. In this study, we aim to detect the internal mechanism of HMGB1 affecting the radio-sensitivity of esophageal cancer cells through PI3K/Akt/ATM pathway.

Previous studies on nasopharyngeal cancer found that patients with high expression of HMGB1 had later tumor stage and poor prognosis after chemo-radiotherapy [28]. Similarly, we found the esophageal squamous cell cancer patients with high expression of HMGB1 showed late TNM stage and short overall survival and progression-free survival in this study, and these were also similar to those of public database TCGA analysis. At the same time, patients with high expression of p-ATM showed late T stage, and prone to relapse which indicate that p-ATM is closely related to the local progression of esophageal cancer. We further analyzed the expression of HMGB1 combined with p-ATM, and the results showed that patients with both positive expression of HMGB1 and p-ATM had larger tumor volume, later T stage, later TNM stage, higher distant metastasis rate, and worse prognosis and survival after chemo-radiotherapy compared with single positive/double negative group. These results suggest that the combination of HMGB1 and p-ATM is a good prognostic indicator for patients with esophageal cancer.

Previous studies have shown that HMGB1 protein is involved in various tumor development processes, especially for proliferation and invasion [29, 30]. In this study, we also found that patients with high expression of HMGB1 had larger tumor volume and later TNM stage. So we looked further into the underlying mechanism. And we found that downregulation of HMGB1 decreased the expression of p-Akt (Ser473) and p-ATM with no change in PI3K, Akt and ATM expression. Activation of PI3K is largely involved in substrates near the inner side of its plasma membrane. Multiple growth factors and signaling complexes, including vascular endothelial growth factor (VEGF), human growth factor (HGF), insulin and IGF1 can initiate PI3K activation, and then activate Akt which is also known as protein kinase B (PKB) or RAS-alpha [31]. Akt is a ubiquitous serine/threonine kinase that plays an important role in diverse biological responses such as regulation of metabolism, cell survival, and growth [32]. This protein kinase is activated by insulin, PI3K and other growth and survival factors. Ly294002 is an inhibitor of PI3 kinase, which normally inhibits Akt activation when PI3 kinase is inhibited. In this study, we use IGF1 and ly294002 as the activator and inhibitor of PI3K/AKT pathway. And we find that knockdown of HMGB1 protein reduced the proliferation and invasion ability of ECA109, which could be reversed by IGF1 with the activation of PI3K/AKT pathway. At the same time, combination of silencing HMGB1 and applying pathway inhibitor ly294002 resulted in the lowest proliferation and invasion of esophageal squamous cancer cells. These findings indicated that HMGB1 may regulate the proliferation and invasion ability of esophageal cancer cells through the PI3K/Akt/ATM signaling pathway. Furthermore, we use flow cytometry to detect the apoptosis and the cell cycle change after downregulation of HMGB1 with or without pathway inhibitor ly294002 or activator IGF1. The results showed the depletion of HMGB1 promoted the apoptosis of esophageal cancer cells induced by IR. But the addition of IGF1 weak the effect of HMGB1 depression on apoptosis increasing of esophageal cancer cells. The cyclin/CDK complexes regulated the cells from transit through G1 of the cell cycle and entry into the S phase, which are predominantly cyclin D1/CDK4 and cyclin E/CDK2. In this research, we verified the effects of HMGB1 expression combined with activator and inhibitor of PI3K/AKT pathway on the transition process of the cell cycle as well. And our findings indicate that the depletion of HMGB1 and the application of PI3K/Akt pathway inhibitors ly294002 have the same effect, both can make cancer cells blocked at G0/G1 stage resulting in apoptosis induction and inhibition of cancer cell proliferation through decreasing the expression of Cyclin D1 and CDK4 and improving P16, however, IGF1 has the opposite effect.

To sum up, activation of the PI3K/Akt pathway reversed radiation sensitization induced by HMGB1 knockdown of esophageal cancer cells. We further validated the cell-level results in animal experiments. And we found the application of HMGB1 silencing, especially combined with pathway inhibitor ly294002, slowed down the growth rate, the tumor volume and weight of xenografts, meanwhile, increased the apoptosis after radiation. Ki67 expression represents the proliferation ability of tumor cells. The expression of p-ATM and Ki67 protein in the xenograft tissue of nude mice was decreased after HMGB1 knockdown, the effect was more pronounced with the application of PI3K/Akt pathway inhibitors ly294002. The above results indicated that the silencing of HMGB1 can inhibit the growth of transplanted tumor, and it does play a radio-sensitization effect in vivo, and its mechanism is related to the PI3K/AKT/ATM signaling pathway.

In conclusion, we found a combination of HMGB1 and p-ATM may predict the prognosis of esophageal cancer patients with chemo-radiotherapy. Downregulation of HMGB1 may promote the radio-sensitivity of esophageal cancer cells through regulating PI3K/Akt/ATM pathway.
